# Toward Morphologically Relevant Extracellular Matrix *in Vitro* Models: 3D Fiber Reinforced Hydrogels

**DOI:** 10.3389/fphys.2018.00966

**Published:** 2018-07-24

**Authors:** Ashok Williams, James F. Nowak, Rachel Dass, Johnson Samuel, K. L. Mills

**Affiliations:** ^1^Department of Mechanical, Aerospace, Nuclear Engineering, Rensselaer Polytechnic Institute, Troy, NY, United States; ^2^Center for Biotechnology and Interdisciplinary Studies, Rensselaer Polytechnic Institute, Troy, NY, United States

**Keywords:** extracellular matrix, electrospinning, hydrogel, mechanobiology, cell-ECM interaction

## Abstract

The extracellular matrix (ECM) is known to play an important role in the health of cells and tissues. Not only are chemical signals transmitted via bonds and tightly controlled diffusion, but the structure of the ECM also provides important physical signaling for the cells attached to it. The structure is composed of a mesh of fibrous proteins, such as collagen, embedded in a hydrated gel matrix of glycosaminoglycans. To study cell behavior with respect to the combined morphology and mechanics of such matrices is not currently possible with the types of 3D cell culture matrices available. Most of the cell culture matrices are single-phase bio- or polymeric hydrogels. Therefore, here we developed a continuous hybrid manufacturing process to make fiber-reinforced composite hydrogels. A far field electrospinning process was used to deposit the fibrous component with the aid of guiding electrodes; and a gravity-assisted, droplet-based system controlled the rate of addition of the cell-laden hydrogel component. The addition of the fibrous component slightly increased the elastic modulus of the pure hydrogel. The cells that were embedded into the fiber-reinforced hydrogels were viable for 8 days. The cells were randomly placed in the matrix such that some had no contact to the fibers and others were initially in proximity to fibers. The cells with no contact to fibers grew into spheroidal clusters within the hydrogel, and those in proximity to the fibers spread out and grew along the fibers showing that the fiber-reinforced hydrogels are able to control cell behavior with morphological cues.

## Introduction

The extracellular matrix (ECM) of connective tissues is composed of two distinctly different morphological components. One of these is a mesh of proteinaceous fibers, such as collagen, fibronectin, and elastin. The other is a highly hydrated gel composed of glycosaminoglycans. Together, they provide structural, biophysical, and biologically active support to the cells within this microenvironment (Theocharis et al., 2016). During the progression of solid tumors, the ECM is altered through increased fibrous protein deposition, reorganization, and cross-linking (Lu et al., 2012). The result is a stiffening, or desmoplasia, of the surrounding tissue (Croft et al., 2004). Pathologically desmoplastic tissues are a major contributor to the biomechanical properties of the tumor microenvironment (Berger et al., 2017). Although this phenomenon is frequently observed, the influence of these mechanical changes on cellular behavior toward the progression of the disease is not fully understood.

Although the mechanobiology of tumor cells is well researched (Makale, 2007), it is often done so in 2D Petri dishes where interactions of the tumor cells with the 3D fiber and gel composite structure of the ECM are ignored. This has lead to a lack of fundamental understanding of the combined effects of matrix stiffening and ECM fiber density on tumor growth. Currently, many investigations utilize reconstituted ECM proteins to mimic the physiology *in vivo* (Narayanan et al., 2009). Although these systems provide 3D microenvironments with native bioactivity, they lack mechanical relevance, as these biomaterials cannot produce scaffolds stiff enough to match relevant physiological or pathophysiological mechanics. Chemical modifications have been used to increase stiffness, but they introduce confounding variables that make it difficult to correctly isolate only the effects of fibers (Erikson et al., 2008). Furthermore, these techniques are unable to control the density of fibrous proteins to correctly isolate their influence on cellular behaviors. Thus, it has been a non-trivial pursuit to produce a system that can capture both morphology and mechanical properties of the evolving tumor microenvironment.

Our main goal, therefore, was to create a 3D *in vitro* ECM model comprised of both fiber and hydrogel components into which cells may be embedded. Furthermore, the fiber density in this model must be readily tunable to capture tissue morphologies from healthy to cancerous. A common method for producing polymeric nano- to micro-scale fibers, often used in cell–material interaction studies, is electrospinning. Typical electrospinning setups produce aligned or random mats of densely packed fibers up to generally a few hundred microns in thickness. Whereas such 2D mats may be used to explore, for example, the contact guidance behavior of cells (Nisbet et al., 2008), they do not capture the 3D nature of the *in vivo* environment. It has proven to be challenging to extend the electrospinning process from easily producing 2D mats to producing 3D fiber networks. Researchers have sought to expand upon the electrospun mats to create hybrid fiber-gel 3D matrices. One method has been to build layered electrospun fiber mat-hydrogel structures (McCullen et al., 2010; Jang et al., 2013; Xu et al., 2013). Briefly, an electrospun mat is covered in a coating of hydrogel and then a second electrospun mat is layered on top and covered in a coating of hydrogel, and so on. This structured approach, however, leads to an imposed layered morphology, which will *a priori* influence the way the cells behave. To our knowledge, only one other research group has attempted to randomly mix electrospun polymeric fibers with a hydrogel matrix. Coburn et al. (2011) report manually collecting fibers that were spun into an organic solvent and mixing them with a poly(ethylene glycol)-diacrylate (PEG-DA) solution before UV exposure to cure the PEG-DA gel.

Here, we report a proof-of-principle study in which we developed a continuous hybrid manufacturing process capable of creating a uniformly random fiber-reinforced hydrogel composite matrix. Without the need for layering or post-electrospinning mixing, we demonstrate the ability to tune the fiber density between samples over a wide range. The process has been designed to incorporate cells into the hydrogel component during production, therefore, fully embedding the cells in the 3D matrix.

## Materials and Methods

### Manufacturing System

**FIGURE 1:**
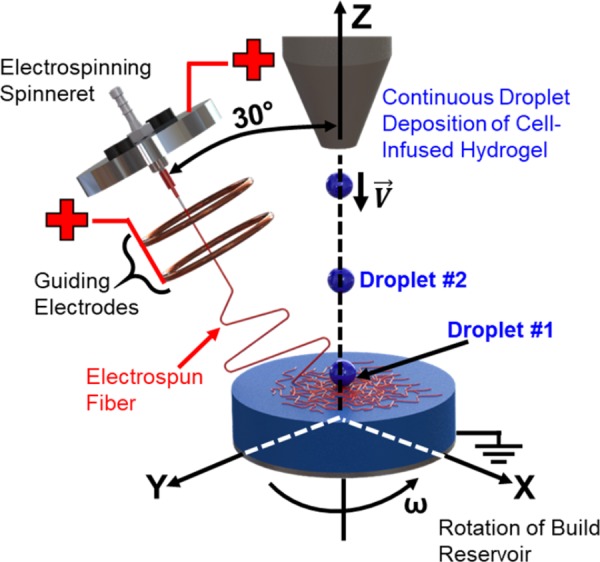
Schematic of the novel hybrid manufacturing process.

In order to create three-dimensional fiber-reinforced hydrogels, as opposed to mats of hydrogel-soaked electrospun fibers, we developed a continuous hybrid manufacturing process (**Figure 1**). The process involves the combination of far-field electrospinning of polymer fiber reinforcement with droplet deposition of the cell-infused hydrogel solution. In order to allow for the combined deposition of both the fiber reinforcement and cell-infused hydrogel droplets, the far-field electrospinning printhead was angled at 30° off of the z-axis, while the path of the cell-infused hydrogel droplets was collinear to the z-axis. In addition, the build reservoir was also rotated about the z-axis. The major premise behind our hybrid manufacturing process is that the wavefront created by the hydrogel droplet impact (Worthington, 1882; Rein, 1993; Manzello and Yang, 2002; Yarin, 2006; Kavehpour, 2015), when combined with rotation of the reservoir, will result in the effective three dimensional distribution of the fibers.

**FIGURE 2:**
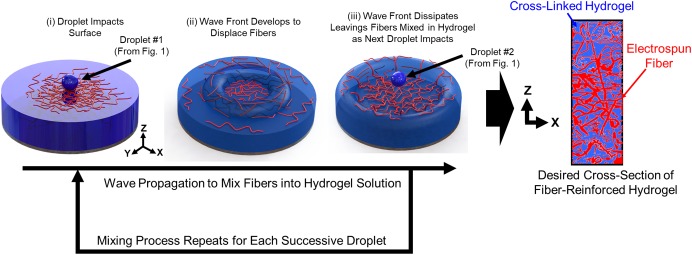
Sequential steps of the droplet impact cycle to create the 3D fiber networks within the hydrogel matrix.

The sequential steps of the droplet impact cycle to create the 3D fiber networks within the hydrogel matrix are as follows (**Figure 2**):

• Step 1: The hydrogel droplet lands on the surface of the build reservoir with its impact velocity.
• Step 2: A wave front is created as the kinetic energy from the droplet is dissipated into the surrounding fluid. The wave front displaces and mixes the electrospun fibers within the three dimensional build volume.
• Step 3: The wave front dissipates, and the next droplet in the sequence proceeds to impact the fluid surface.
• Step 4: Steps 1–3 are repeated until the desired thickness of the sample is achieved.
• Step 5: Upon gelation, the cross-section of the fiber network is characterized.

It should be noted the rotational motion of the build reservoir augments the mechanisms at play in Steps 2 and 3. This additional energy not only mixes the electrospun fibers in the fluid (Step 2) but it also introduces a new deposition zone for the eletrospun fibers and hydrogel solution (Step 3).

**FIGURE 3:**
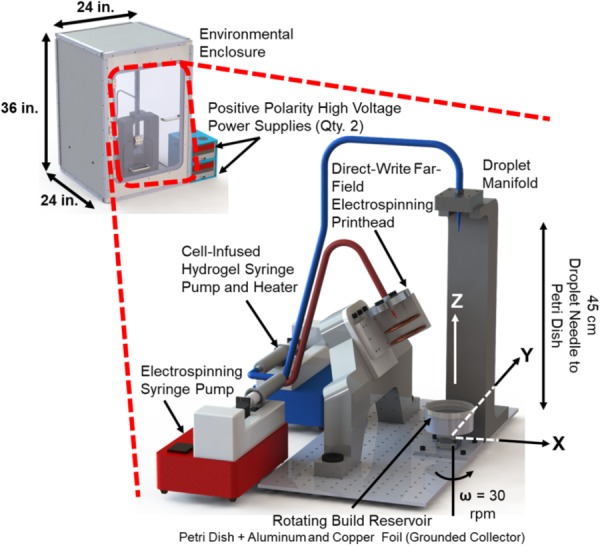
Implementation of the hybrid manufacturing system for cell-infused fiber reinforced hydrogel production.

The components of the manufacturing system include the far-field electrospinning print head the electrospinning solution syringe pump (R99, Razel, St. Albans, VT, United States), two positive-polarity, high-voltage power supplies (ES-50P, Gamma High Voltage, Ormond Beach, FL, United States), the hydrogel dispensing printhead, the hydrogel solution syringe pump (R99, Razel, St. Albans, VT, United States) with syringe heating pad and controller (New Era Pump Systems, Farmingdale, NY, United States), and build reservoir on a rotary stepper motor stage (maximum rotational speed of 30 revolutions per minute). The manufacturing system was placed inside of an environmental enclosure (**Figure 3**) constructed from polypropylene, due to its high electrical resistivity and chemical resistance. The following section describes the components and process methodology of the system in detail.

#### Electrospinning Process

The electrospinning process utilized a far-field setup, with guiding electrodes located underneath the spinneret needle. The spinneret design consists of a round, aluminum-6061-T6 alloy manifold (where the polymer solution was fed from a syringe pump). The spinneret voltage was applied to the manifold via a positive-polarity, high-voltage power supply in order to charge the polymer solution and blunt-ended needle (21-gauge by 12 mm long, stainless steel). The round shape of the manifold was selected so that it would better transmit the electric field to the collection substrate.

**Table 1:** Electrospinning process parameters.

Spinneret voltage	+16–19 kV
Guiding electrode voltage	+9–10 kV
Stainless steel needle size	820 μm (21 G)
Solution feed rate	0.1 mL/min
Spinneret distance	25–30 cm

The use of guiding electrodes has been used in previous electrospinning experiments to reduce the spread of the fiber deposition (Deitzel et al., 2001; Lee et al., 2012; Martinez-Prieto et al., 2015). With the addition of these guiding electrodes (i.e., copper rings), a second positive-polarity, high-voltage power supply was required to allow for the independent control of both the spinneret voltage (applied to the needle), and the focusing electrode voltage (applied to the rings). Here, the guiding electrodes were two solid copper rings (rolled diameter 75 mm, tube diameter of 4 mm), mounted concentric to the manifold and needle assembly. The rings were rolled to a ring diameter of 75 mm. The two copper rings were located 40 and 60 mm, respectively, below the bottom surface of the manifold.

The electrospinning process parameters used in this setup are listed in **Table 1**. The ground electrode, required for electrospinning, was a round sheet of aluminum foil affixed to the bottom of the build reservoir (a 100 mm diameter polystyrene Petri dish).

#### Hydrogel Dispensing

The hydrogel dispensing system was a gravity-assisted, droplet-based deposition system. The hydrogel solution was supplied to a dispensing head located directly above the build reservoir through the use of a syringe pump. The syringe pump supplied pressure to force a droplet to grow at the tip of a tapered polypropylene dispensing needle (14-gauge, 1.6 mm inside diameter). The height at which the hydrogel dispensing head was located above the build reservoir dictated the velocity (and energy) of the impacting droplet. This was set constant at a height of 45 cm above the build reservoir, which resulted in a droplet velocity of approximately 3 m/s. When embedding cells, they were suspended in the hydrogel at 40°C (see section “Hydrogel” below) and aspirated into the syringe just before the manufacturing process began. The cell-infused hydrogel solution was maintained at a temperature of 40°C in the syringe using the syringe heater. The entire manufacturing process for one sample lasts approximately 3 min.

### Electrospinning and Hydrogel Materials

#### Electrospinning Solution

Polycaprolactone (PCL) (*M*_n_ = 80,000, Sigma-Aldrich, St. Louis, MO, United States) was used as the material for the electrospun fiber-reinforcement. A concentration of 17 wt.%. was dissolved in glacial acetic acid (Sigma-Aldrich, St. Louis, MO, United States). While being biocompatible, PCL suffers from exhibiting hydrophobic properties, which can inhibit cell adhesion (Liverani and Boccaccini, 2016; Mirhosseini et al., 2016). In order to improve these wetting properties, pluronic acid F-127 (concentration 1.0 wt.%) was added to the electrospinning solution. The solution was mixed for 12–18 h at 60°C.

#### Hydrogel

Low gelling temperature agarose (Sigma-Aldrich, St. Louis, MO, United States) was dissolved in incomplete McCoys 5A (Gibco) cell culture medium. For these experiments, the concentration of agarose used was 0.3% w/v to simulate the native ECM stiffness of the cells being used (Mills et al., 2011, 2014; Yu et al., 2011). When creating cell-laden matrices, cells were mixed into the agarose solution at a density of 80,000 cells/mL immediately prior to deposition. After the agarose was deposited, the sample was placed in the refrigerator (4°C) for 30 min to hasten gelation. After gelling, the sample was washed in incomplete medium three times for 5 min. The incomplete medium was then replaced with supplemented medium and the sample placed in the incubator. The washing steps removed traces of the solvent needed to create the fibers and brought the sample to physiological pH.

### Cell Culture and Staining

Human colon cancer cells (HCT 116) were embedded in the fiber-reinforced hydrogels. Once cells were embedded, the samples were hydrated with McCoys 5A cell culture medium supplemented with 10% fetal bovine serum, and 1% penicillin, streptomycin, and amphotericin. Samples were incubated at 37°C and 5% CO_2._ The cell-culture medium was changed every 48 h.

After 8 days of incubation and observation, cell-laden fiber-reinforced hydrogel samples were fixed and stained for fluorescence imaging with the confocal microscope. First, the samples were washed three times for 5 min each in phosphate buffered saline (PBS). They were then fixed overnight at 4°C in 4% paraformaldehyde. After three more 5 min washes in PBS, the samples were permeabilized in 0.2% Triton X-100 detergent for 30 min followed by three further 5 min washes in PBS. The samples were blocked in 1% bovine serum albumin solution for 30 min before incubating in rhodamine phalloidin (1:75 dilution in PBS) to stain F-actin. The nuclei were then stained with Hoechst (0.2 μg/mL, Hoechst 33342, Thermo Fisher) at room temperature in dark for 4 h.

### Microscopy

#### For Fiber Characterization

To obtain clean cross-sections for imaging, the fiber-reinforced hydrogel samples were dehydrated and cooled prior to sectioning. The samples were dehydrated in a series baths, of increasing ethanol concentration, over a period of 24 h. The sample was first placed in a 33% ethanol bath for 8 h, a 66% ethanol bath for 8 h, and finally in a 100% ethanol bath for 8 h. Then, the samples were cooled in a refrigerator (2–4°C) for 12 h. Finally, the samples were mechanically sectioned using a tungsten-carbide cutter. The cross-sections of these samples were imaged using an optical microscope (Zeta 20, Zeta Instruments, San Jose, CA, United States), at a magnification of 50×. Cross-sectional images were then analyzed in ImageJ image analysis software to measure the fiber diameter distribution, and fiber number density of the produced samples.

#### For Cell Morphological Characterization

For the cell-incorporated fiber-reinforced hydrogels, an inverted optical microscope (Axio Vert.A1, Zeiss, Jena, Germany) was used to take images daily. Ten images were captured per sample in random regions, while making sure to document cell behavior near or on fibers and away from fibers. Fluorescence images of cells and fibers were acquired with a laser scanning confocal microscope (LSM 510 Meta, Zeiss, Jena, Germany).

### Mechanical Characterization

**FIGURE 4:**
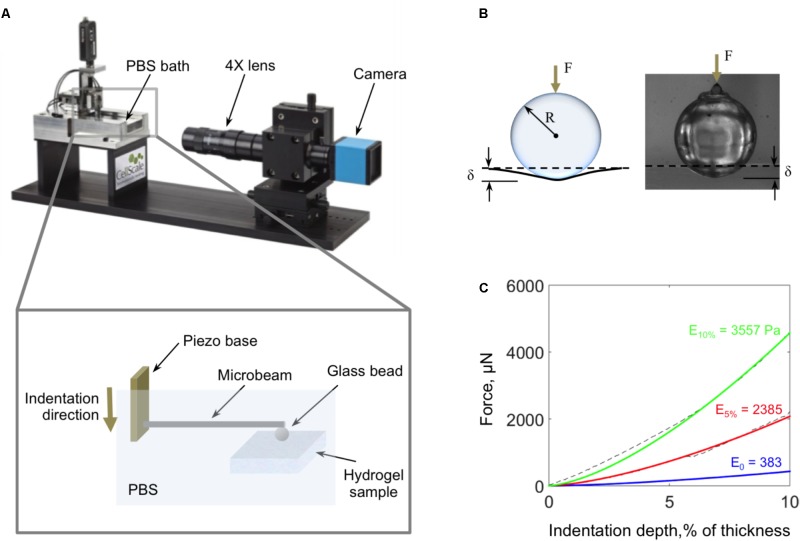
**(A)** Schematic of the micro-indentation system used to characterize the mechanical properties of the fiber-reinforced hydrogels. **(B)** Schematic and image of the 3 mm diameter glass bead and the view of the camera as it indents the hydrogel surface. **(C)** Samples of characteristic force-indentation depth curves (dashed lines) and their corresponding Hertz equation fits for fiber-reinforced hydrogels with 10% fiber concentration (green), 5% fiber concentration (red), and pure agarose (blue).

Micro-indentation tests were performed using a high-precision piezo-electric actuator controlled microcompression system (CellScale Biomaterials Testing, Waterloo, ON, Canada; **Figure 4A**). The hydrogels were indented with a 3 mm glass bead, which was glued to the end of cantilevered steel microbeams of 0.2032 or 0.4064 mm diameter (**Figure 4B**). Indentation force, *F*, and depth, δ, were continuously calculated during the experiment based on the deflection of the indenter end of the cantilevered beam (measured optically) and the piezo-controlled z-displacement of the cantilevered beam’s fixed end. Experimental force-indentation depth curves were fit to determine the sample’s elastic modulus, *E*, using the Hertz contact model for a spherical indenter (Yang et al., 2007):

$$F=\frac{4}{3}\frac{ER^{1/2}}{3(1-v^{2})}\delta^{3/2}$$

where *R* is the radius of the indenter and ν is the Poisson’s ratio, which was assumed to be 0.49 for the hydrogels (**Figure 4C**).

When testing, fiber-reinforced hydrogel samples were kept in 60 mm Petri dishes with the walls carefully removed, and the entire sample and microbeam were submerged in PBS. The indentation and retraction rates were the same and kept constant between tests at 4 μm/s. The maximum indentation depth was 10% of the height of the sample.

## Results

### Fiber-Reinforced Hydrogels Microscale Morphology

**FIGURE 5:**
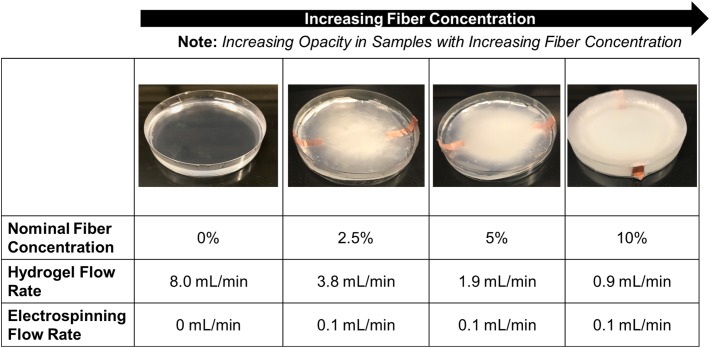
Representative images of the fiber-reinforced hydrogels with varying fiber concentration (Petri dish has a diameter of 100 mm).

The use of a syringe pump allows for the volumetric flow rate of the hydrogel solution to be tailored to allow for varying fiber densities in the final fiber-reinforced hydrogel samples. Varying the volumetric flow rate of the hydrogel solution between 3.8 and 0.9 mL/min, with the electrospinning rate held constant at 0.1 mL/min, we observed nominal fiber densities between 2.5 and 10%, respectively (**Figure 5**).

**FIGURE 6:**
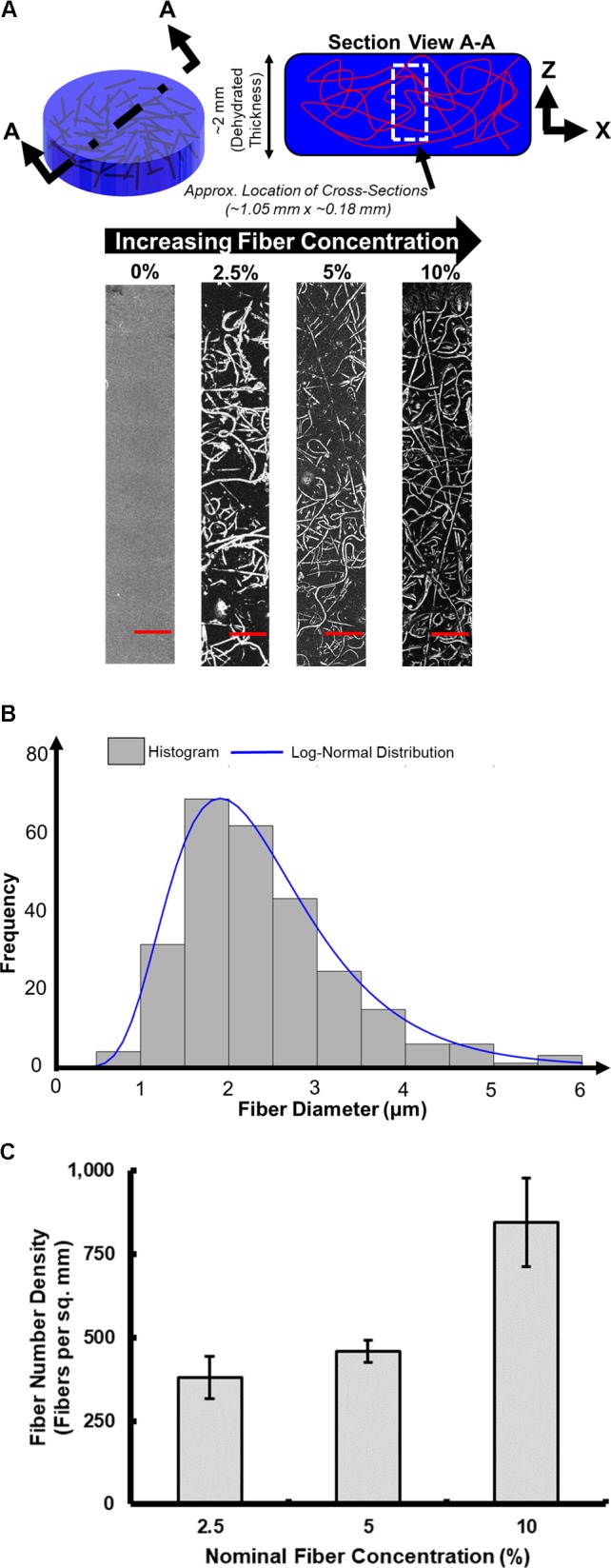
Optical microscopy characterization of fiber-reinforced hydrogels. **(A)** Characteristic cross-sectional images (scale bar is 100 μm). **(B)** Distribution of measured fiber diameters. **(C)** Fiber number density.

Representative samples at the different fiber densities were selected for cross-sectioning and imaging (**Figure 6A**). From these images we observed the morphology of the fibrous network and whether it was deposited uniformly through the thickness of the sample, we also measured the fiber diameter (**Figure 6B**) and fiber number density (**Figure 6C**). Visually we observe (**Figure 6A**) that the fibrous network uniformly penetrates the full imaged depth of the samples. The network appears to be comprised of randomly oriented fibers, both in-plane (observed as bright lines) and out-of-plane (observed as bright dots).

**Figure 6B** displays a histogram of the measured fiber diameters pooled across all the samples, consisting of varying fiber concentrations produced (*n* = 9 samples). The distribution of fiber diameter followed a lognormal distribution, with a mean and standard deviation of 2.40 and 0.98 μm, respectively. No significant changes in fiber diameter were seen across samples with different fiber concentrations, as the electrospinning parameters (listed in **Table 1**), and electrospinning solution (detailed in section Electrospinning Solution) remained constant throughout the production of all samples.

The fiber number density was also computed for the samples produced at different fiber concentrations. **Figure 6C** presents value of the fiber number density (i.e., number of fibers per unit area), seen in the optical microscopy images. A total of three optical microscopy images were analyzed for each fiber concentration on the plot. As the fiber concentration increases, the fiber number density also increases. Using this process, the fiber number density increase up to ∼850 fibers per sq. mm at the highest fiber concentration (10%) that was created.

### Mechanical Characterization of the Fiber-Reinforced Hydrogels

**FIGURE 7:**
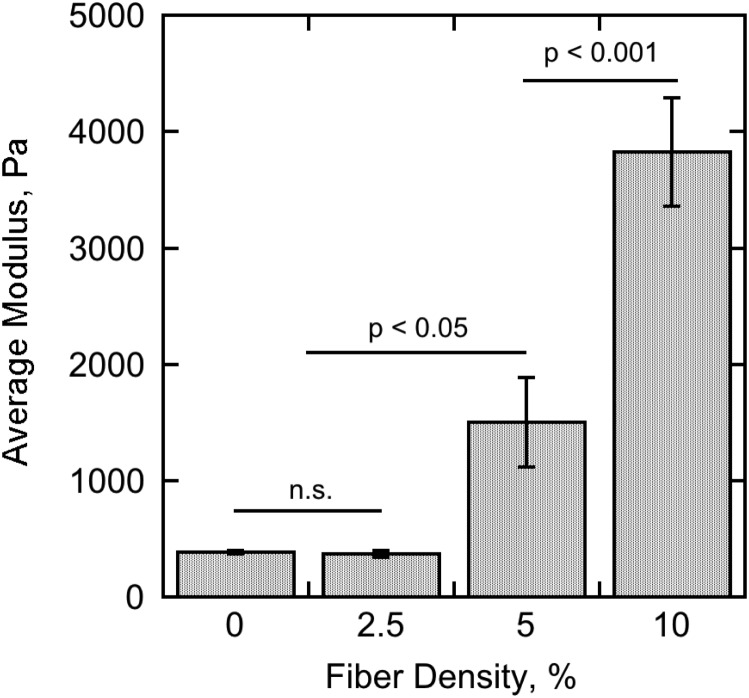
Summary of the micro-indentation experimental results for pure 0.3% agarose hydrogel (0% nominal fiber concentration) and 0.3% agarose hydrogel reinforced with 2.5, 5, and 10% nominal fiber concentration. The bar heights represent the average elastic modulus calculated from the Hertz contact equation fit to at least four indentation measurements on at least three separate samples for each condition. The error bars represent the standard error of the measurements. Significance was tested using one-way ANOVA with Tukey’s *post hoc* test for multiple comparisons.

At least three samples were produced of pure 0.3% agarose hydrogel and fiber-reinforced hydrogels with fiber densities of 2.5, 5, and 10% for mechanical testing by micro-indentation. The elastic moduli for pure 0.3% agarose and 2.5% fiber-reinforced hydrogel were found to be 385 ± 13 Pa and 368 ± 28 Pa, respectively, and not significantly different from one another. Upon the incorporation of higher concentrations of fibers, however, the elastic modulus rose to 1,500 ± 387 Pa with 5% fiber concentration and 3,830 ± 466 Pa with 10% fiber concentration (**Figure 7**). At least four indentation measurements per sample (12 total per condition) were collected for the averages and standard errors presented here.

### Cell Growth and Morphological Characterization

**FIGURE 8:**
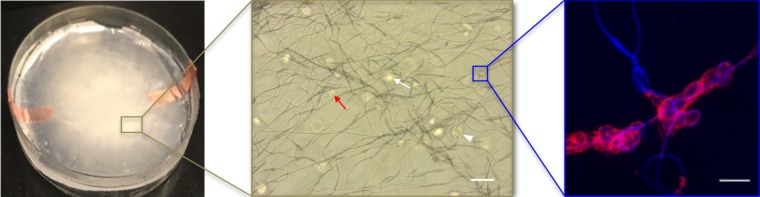
The fiber reinforced hydrogel appearance at macro-scale (left, 100 mm diameter Petri dish) and at increasingly smaller micro-scales (middle, scale bar 100 μm; right, scale bar 50 μm). In the middle image, cell clusters appear in focus (white arrow head) or out of the focus plane (white arrow); oddly shaped cell clusters at this magnification often indicate cell attachment to fibers (red arrow). In the right image, a higher-magnification, confocal image of cells attached to fibers at a junction where fibers cross (red is F-actin, blue is autofluorescence of the fibers).

**FIGURE 9:**
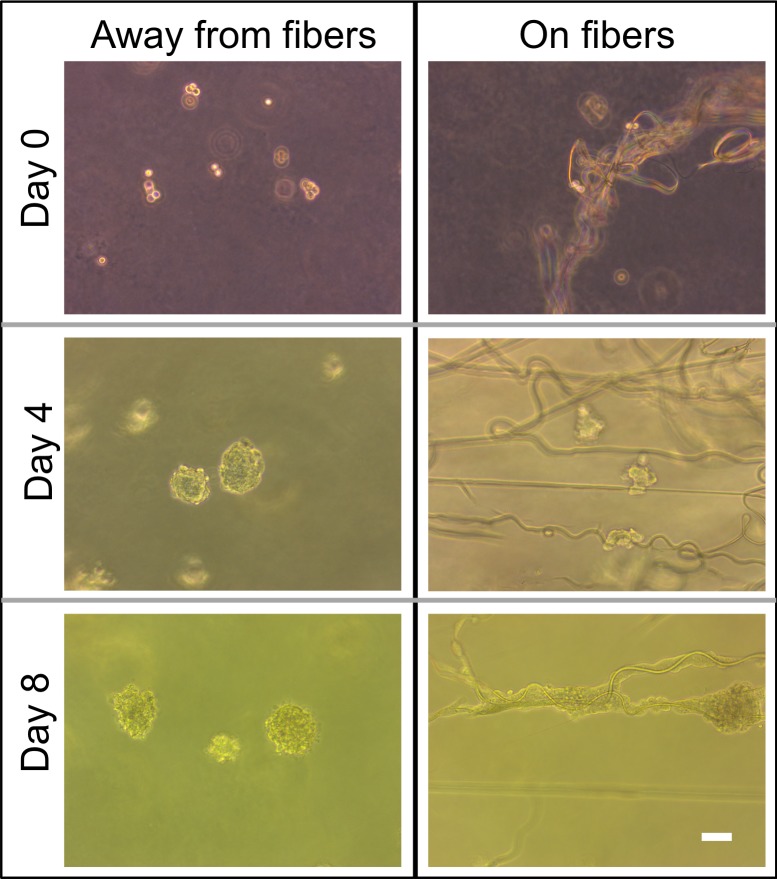
Comparison of phase contrast images of cell morphology away from fibers and on fibers directly after seeding on day 0 and on days 4 and 8. Scale bar is 50 μm.

**FIGURE 10:**
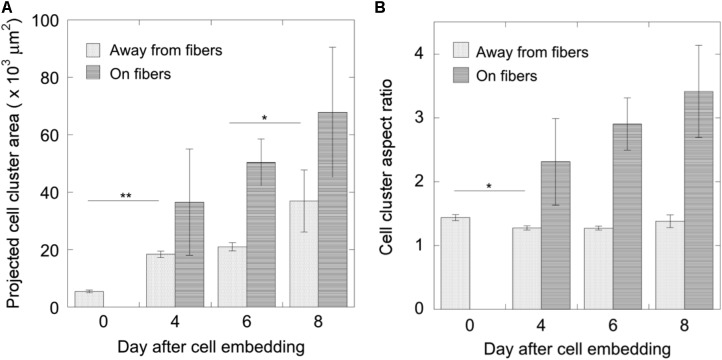
Measurements of cell cluster development over 8 days. **(A)** The growth of cell clusters was quantified as their projected area and **(B)** their morphology as their aspect ratio (mean ± standard error). For cell clusters growing away from fibers, *N*_0_ = 35, *N*_4_ = 43, *N*_6_ = 27, and *N*_8_ = 12; for cell clusters growing on fibers, *N*_4_ = 3, *N*_6_ = 4, *N*_8_ = 7. Significance was tested using one-way ANOVA with Tukey’s *post hoc* test for multiple comparisons (^∗^*p* < 0.05, ^∗∗^*p* < 0.001).

**FIGURE 11:**
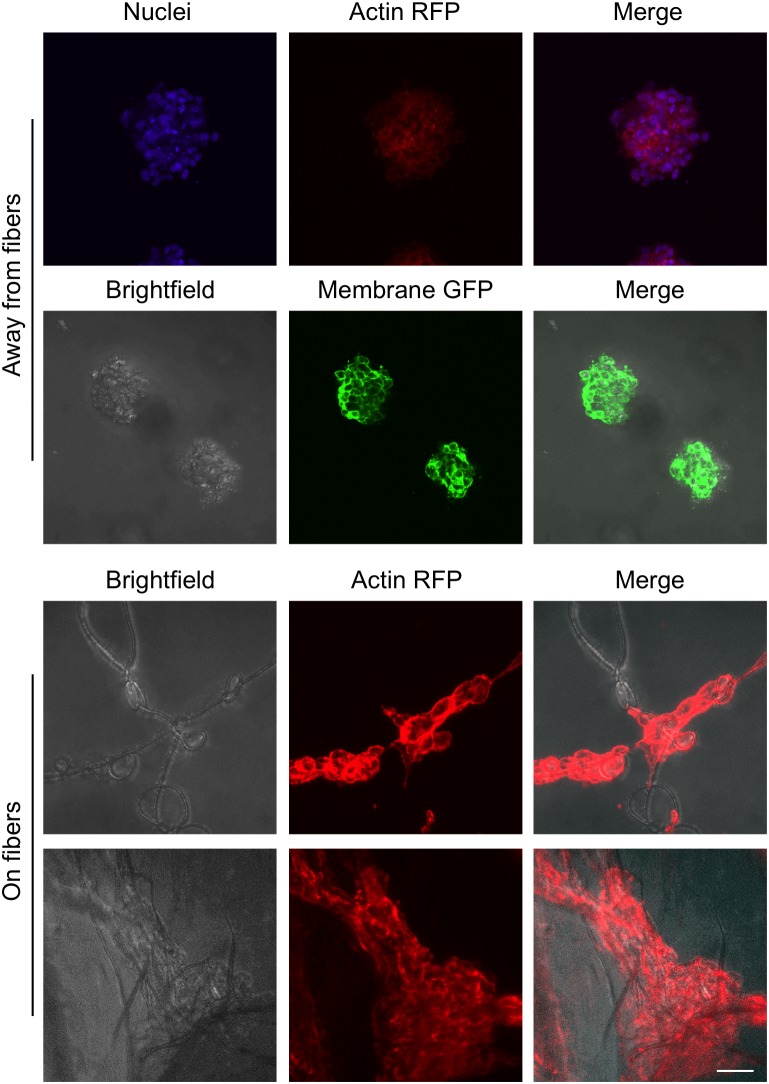
Images of cell clusters growing away from fibers and on fibers. The brightfield, nuclei (Hoechst, blue), membrane (GFP, green), and actin (RFP phalloidin, red) images are the maximum projection of z-stacks of images taken with a confocal microscope. Scale bar is 50 μm.

For this proof of principle study we chose an approximately 4% fiber concentration as we found this was an optimal density that maximized the probability of cell–fiber interaction while maintaining optical transparency of the matrix to obtain microscope images of the cells (**Figure 8** and Supplementary Figure 1). In order to initiate cell-fiber contact, a cluster of cells must be embedded in proximity to a fiber or bundle of fibers. Growth and morphology of the embedded HCT-116 cell clusters were monitored over an 8 days period in the fiber-reinforced hydrogels (**Figures 9**, **10**). Characteristic images of cell clusters growing away from and on the fibers within the hydrogels show the obvious effects of the fibers on growth morphology (**Figures 9**, **11**). We quantified the growth rates of the cell clusters as their projected area in the micrographs and their morphology as their projected aspect ratio over 8 days of growth (**Figure 10**). Initially (day 0), the embedded cell clusters are not apparently attached to fibers; therefore, no data is recorded for that day and condition. Cell clusters that are away from fibers and on fibers both exhibit steady growth (**Figure 10**, left). The most significant effect of the presence of the fibers on cell growth, however, is the morphology. Whereas cell clusters away from the fibers maintain an approximately spherical morphology (**Figure 10**, right; **Figure 11**, top rows), the clusters on fibers extend out along the fibers and project a more elongated shape over time (**Figure 10**, right; **Figure 11**, bottom rows). Since the probability of a cell cluster being embedded in contact with a fiber is relatively low, there are significantly fewer measurements for “on fiber” clusters as compared to “away from fibers” clusters. After the 8 days growth period, the samples were fixed and stained with either Hoechst (to visualize the nuclei) or RFP phalloidin (to visualize the F-actin network). Although the characterization of cell growth and morphology has been presented here for the HCT-116 colon cancer cell line, the applicability of the method to a second cell line (MDA-MB-231 breast adenocarcinoma) is presented in the Supplementary Materials (Supplementary Figure 2).

## Discussion

Morphological guidance cues play an important role in determining cell behavior, from spreading and proliferation to apoptosis. It has been recognized for some time the need to culture cells in environments that more closely resemble their native 3D, soft, and structured ECM than 2D, stiff Petri dish plastic. However, the commonly used bio- and synthetic polymeric hydrogels lack the important protein fiber structures that are present in the body. Furthermore, the protein fiber structure is known to become significantly denser and stiffer due to increased deposition and cross-linking in diseases like fibrosis and cancer. Since no *in vitro* cell culture matrix is available that combines both the fibrous and gel components of the ECM, nor the ability to tune the fiber density and mechanical properties of the matrix, our major goal here was to produce such a matrix and demonstrate cell viability and morphological differences in it.

Here, we developed a continuous hybrid manufacturing process to make fiber-reinforced composite hydrogels. A far field electrospinning process was used to deposit the fibrous component with the aid of guiding electrodes; and a gravity-assisted, droplet-based system controlled the rate of addition of the cell-laden hydrogel component. An environmental enclosure was designed in which to carry out the manufacturing of the samples to avoid contamination. For these proof of principle experiments, we used PCL for the electrospun fibers and 0.3% agarose for the gel matrix due to their ease of use and cost effectiveness, but other commonly electrospun materials and hydrogels may also be implemented with little effort.

Cells were introduced into the fiber-reinforced hydrogels by suspending them in the hydrogel solution prior to its droplet deposition. The cells were therefore randomly placed in the matrices, sometimes lying near to or on fibers and sometimes away from fibers. We tracked how the cells grew and responded with respect to their position in the gels. Obvious differences could be seen between cells that were growing away from fibers, and cells growing along fibers. Such distinctive phenotypic differences were expected. Agarose is a bioinert, non-cell-adhesive hydrogel that only provides tissue-mechanics-mimicking structural support for cell growth. Therefore, it was not surprising that cell clusters that were only in contact with this gel maintained a compacted spheroidal shape. The use of other, bioactive hydrogels would add another layer of complexity to this system. With little to no modification of the manufacturing system, other thermo-sensitive gelling materials may be used (e.g., gelatin; Supplementary Figure 3). Cells that were embedded near to or in contact with the PCL fibers, however, displayed a completely different phenotype. They attached to the PCL fibers, spreading out along them in long thin bundles. This demonstrated the ability to control cell behavior with a randomly embedded, 3D polymeric fiber mesh embedded in a hydrogel matrix. This will be a useful tool to study how cells interact with fibrotic and desmoplastic tissues, which are known to be part of the pathophysiology of tumor growth and metastasis. Excessive fibrous structures in and surrounding the tumor are known, for example, to provide highways upon which tumor cells migrate (Condeelis and Segall, 2003).

Continuous improvement of this manufacturing process will allow greater control over fiber density, and possibly fiber orientations, within the hydrogel matrix. Such fully tunable matrices will provide mechanobiologists a useful tool with which to study the interactions of cells with the ECM and determine their influence in health and disease.

## Author Contributions

AW performed cell experiments, analyzed data, and wrote sections of the manuscript. JN designed and built the hybrid manufacturing process, characterized the fiber morphology of the samples, and wrote sections of the manuscript. RD developed the microindentation system and mechanically characterized the samples. JS guided the efforts related to the development of the hybrid manufacturing process and the subsequent fiber characterization. KM conceived and contributed to the design of the study, interpreted the results, and wrote sections of the manuscript. All authors contributed to manuscript revision, read, and approved the submitted version.

## Conflict of Interest Statement

The authors declare that the research was conducted in the absence of any commercial or financial relationships that could be construed as a potential conflict of interest. The reviewer PM and handling Editor declared their shared affiliation.

## References

[B1] Berger A. J. Linsmeier K. M. Kreeger P. K. Masters K. S. (2017). Decoupling the effects of stiffness and fiber density on cellular behaviors via an interpenetrating network of gelatin-methacrylate and collagen. *Biomaterials* 141 125–135. 10.1016/j.biomaterials.2017.06.039 28683337 PMC5556948

[B2] Coburn J. Gibson M. Bandalini P. A. Laird C. Mao H. -Q. Moroni L. (2011). Biomimetics of the Extracellular matrix: an integrated three-dimensional fiber-hydrogel composite for cartilage tissue engineering. *Smart Struct. Syst.* 7 213–222. 10.12989/sss.2011.7.3.213 22287978 PMC3266370

[B3] Condeelis J. Segall J. E. (2003). Intravital imaging of cell movement in tumours. *Nat. Rev. Cancer* 3 921–930. 10.1038/nrc1231 14737122

[B4] Croft D. R. Sahai E. Mavria G. Li S. Tsai J. Lee W. M. F. (2004). Conditional ROCK activation in vivo induces tumor cell dissemination and angiogenesis. *Cancer Res.* 64 8994–9001. 10.1158/0008-5472.CAN-04-2052 15604264

[B5] Deitzel J. M. Hirvonen J. K. Beck Tan N. C. Kleinmeyer J. D. (2001). Controlled deposition and collection of electro-spun poly (ethylene oxide) fibers. *Polymer* 42 8163–8170. 10.1016/S0032-3861(01)00336-6

[B6] Erikson A. Andersen H. N. Naess S. N. Sikorski P. de Lange Davies C. (2008). Physical and chemical modifications of collagen gels: impact on diffusion. *Biopolymers* 89 135–143. 10.1002/bip.20874 17957715

[B7] Jang J. Lee J. Seol Y. J. Jeong Y. H. Cho D. W. (2013). Improving mechanical properties of alginate hydrogel by reinforcement with ethanol treated polycaprolactone nanofibers. *Compos. B Eng.* 45 1216–1221. 10.1016/j.compositesb.2012.09.059

[B8] Kavehpour H. P. (2015). Coalescence of drops. *Annu. Rev. Fluid Mech.* 47 245–268. 10.1146/annurev-fluid-010814-014720

[B9] Lee J. Lee S. Y. Jang J. Jeong Y. H. Cho D. W. (2012). Fabrication of patterned nanofibrous mats using direct-write electrospinning. *Langmuir* 28 7267–7275. 10.1021/la3009249 22512407

[B10] Liverani L. Boccaccini A. R. (2016). Versatile production of poly(Epsilon-Caprolactone) fiber by electrospinning using benign solvents. *Nanomaterials* 6 15. 10.3390/nano6040075 28335202 PMC5302571

[B11] Lu P. Weaver V. M. Werb Z. (2012). The extracellular matrix: a dynamic niche in cancer progression. *J. Cell Biol.* 196 395–406. 10.1083/jcb.201102147 22351925 PMC3283993

[B12] Makale M. (2007). Cellular mechanobiology and cancer metastasis. *Birth Defects Res. C Embryo Today Rev.* 81 329–343. 10.1002/bdrc.20110 18228263

[B13] Manzello S. L. Yang J. C. (2002). An experimental study of a water droplet impinging on a liquid surface. *Exp. Fluids* 32 580–589. 10.1007/s00348-001-0401-8

[B14] Martinez-Prieto N. Abecasis M. Xu J. Guo P. Cao J. Ehmann K. F. (2015). Feasibility of fiber-deposition control by secondary electric fields in near-field electrospinning. *J. Micro Nanomanuf.* 3:6 10.1115/1.4031491

[B15] McCullen S. D. Miller P. R. Gittard S. D. Gorga R. E. Pourdeyhimi B. Narayan R. J. (2010). In Situ collagen polymerization of layered cell-seeded electrospun scaffolds for bone tissue engineering applications. *Tissue Eng. C Methods* 16 1095–1105. 10.1089/ten.tec.2009.0753 20192901

[B16] Mills K. L. Garikipati K. Kemkemer R. (2011). Experimental characterization of tumor spheroids for studies of the energetics of tumor growth. *Int. J. Mater. Res.* 102 889–895. 10.3139/146.110532

[B17] Mills K. L. Kemkemer R. Rudraraju S. Garikipati K. (2014). Elastic Free energy drives the shape of prevascular solid tumors. *PLoS One* 9:e103245. 10.1371/journal.pone.0103245 25072702 PMC4114546

[B18] Mirhosseini M. M. Haddadi-Asl V. Zargarian S. S. (2016). Fabrication and characterization of hydrophilic poly(ε-caprolactone)/pluronic P123 electrospun fibers. *J. Appl. Polym. Sci.* 133:11 10.1002/app.43345

[B19] Narayanan K. Leck K. J. Gao S. Wan A. W. (2009). Three-dimensional reconstituted extracellular matrix scaffolds for tissue engineering. *Biomaterials* 30 4309–4317. 10.1016/j.biomaterials.2009.04.049 19477508

[B20] Nisbet D. R. Forsythe J. S. Shen W. Finkelstein D. I. Horne M. K. (2008). Review paper: a review of the cellular response on electrospun nanofibers for tissue engineering. *J. Biomater. Appl.* 24 7–29. 10.1177/0885328208099086 19074469

[B21] Rein M. (1993). Phenomena of liquid drop impact on solid and liquid surfaces. *Fluid Dyn. Res.* 12 61–93. 10.1016/0169-5983(93)90106-K

[B22] Theocharis A. D. Skandalis S. S. Gialeli C. Karamanos N. K. (2016). Extracellular matrix structure *Adv. Drug Deliv. Rev.* 97 4–27. 10.1016/j.addr.2015.11.001 26562801

[B23] Worthington A. M. (1882). On impact with a liquid surface. *Proc. R. Soc. Lond.* 34 217–230. 10.1098/rspl.1882.0035

[B24] Xu T. Binder K. W. Albanna M. Z. Dice D. Zhao W. Yoo J. J. (2013). Hybrid printing of mechanically and biologically improved constructs for cartilage tissue engineering applications. *Biofabrication* 5:015001. 10.1088/1758-5082/5/1/015001 23172542

[B25] Yang Y. Bagnaninchi P. O. Ahearne M. Wang R. K. Liu K.-K. (2007). A novel optical coherence tomography-based micro-indentation technique for mechanical characterization of hydrogels. *J. R. Soc. Interface* 4 1169–1173. 10.1098/rsif.2007.1044 17472904 PMC2396212

[B26] Yarin A. L. (2006). Drop impact dynamics: splashing, spreading, receding, bouncing. *Annu. Rev. Fluid Mech.* 38 159–192. 10.1146/annurev.fluid.38.050304.092144

[B27] Yu H. Mouw J. K. Weaver V. M. (2011). Forcing form and function: biomechanical regulation of tumor evolution. *Trends Cell Biol.* 21 47–56. 10.1016/j.tcb.2010.08.015 20870407 PMC3014395

