# Supplementary material for: Toward Morphologically Relevant Extracellular Matrix in Vitro Models: 3D Fiber Reinforced Hydrogels
Source: Front Physiol. 2018 Jul 24;9:966. doi: 10.3389/fphys.2018.00966 (PMC6066552; doi:10.3389/fphys.2018.00966)
Supplement: Supplementary file 1 [file Data_Sheet_1.DOCX]

Supplementary Material

Towards Morphologically Relevant Extracellular Matrix In Vitro Models: 3D Fiber Reinforced Hydrogels

Ashok Williams^1,2†^, James F. Nowak^1†^, Rachel Dass^1,2^, Johnson Samuel^1^, K.L. Mills^1,2*^

*** Correspondence:** Corresponding Author: millsk2@rpi.edu

# Supplementary Materials and Methods

Human breast adenocarcinoma cells (MDA-MB-231) were cultured in RPMI 1640 (Sigma), supplemented with 2mM L-glutamine (Sigma), 5% FBS (Gibco), and 1X PSA (Sigma). Embedding and staining protocols follow those in Section 2.3 of the main paper.

# Supplementary Figures


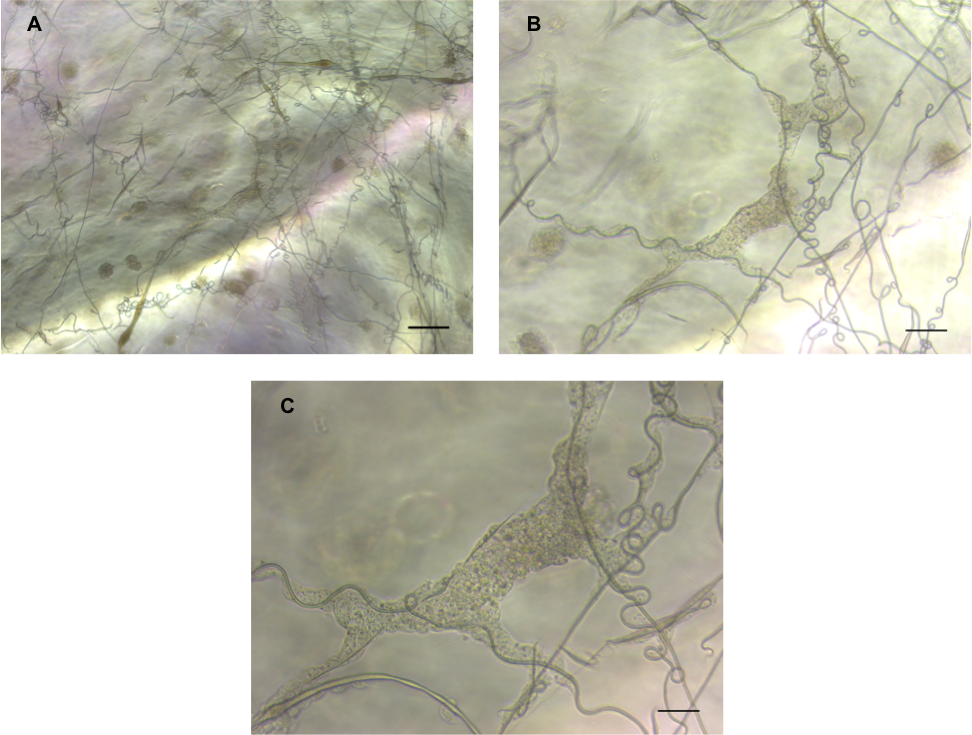


**Supplementary Figure 1.** The appearance of the cell cluster-laden fiber-reinforced hydrogels at different magnifications. Images were taken with a (A) 5x objective (scale bar is 200 μm), a (B) 10x objective (scale bar is 100 μm), and a (C) 20x objective (scale bar is 50 μm). In each successive increase in magnification, it is the center region of the image—where a cluster of cells has spread between at least two junctions of fibers—that is being captured.


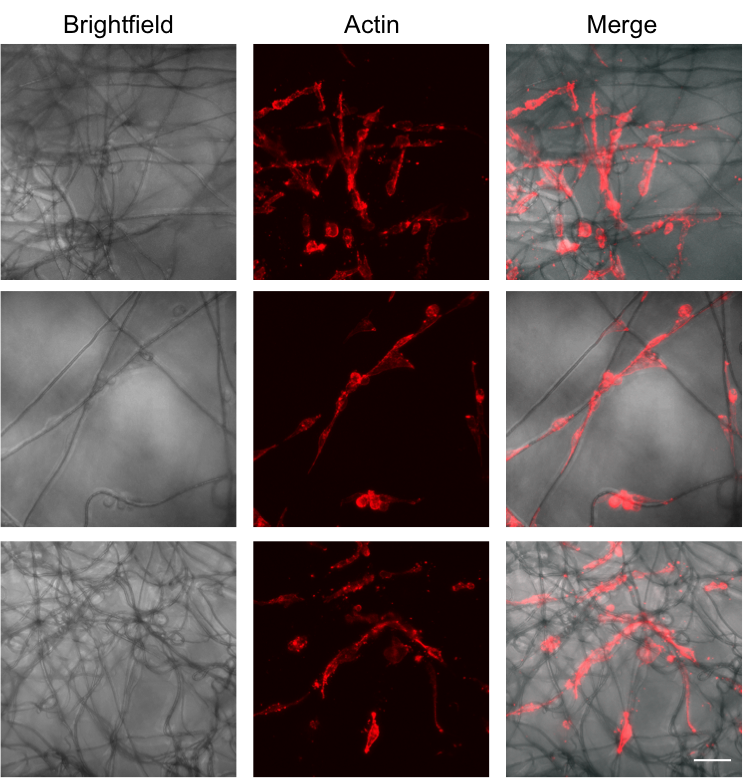


**Supplementary Figure 2.** Human breast adenocarcinoma cells (MDA-MB-231) growing along fibers within agarose hydrogels. The brightfield and actin (RFP phalloidin, red) images are the maximum projection of z-stacks of images taken with a confocal microscope. Scale bar is 50 μm.


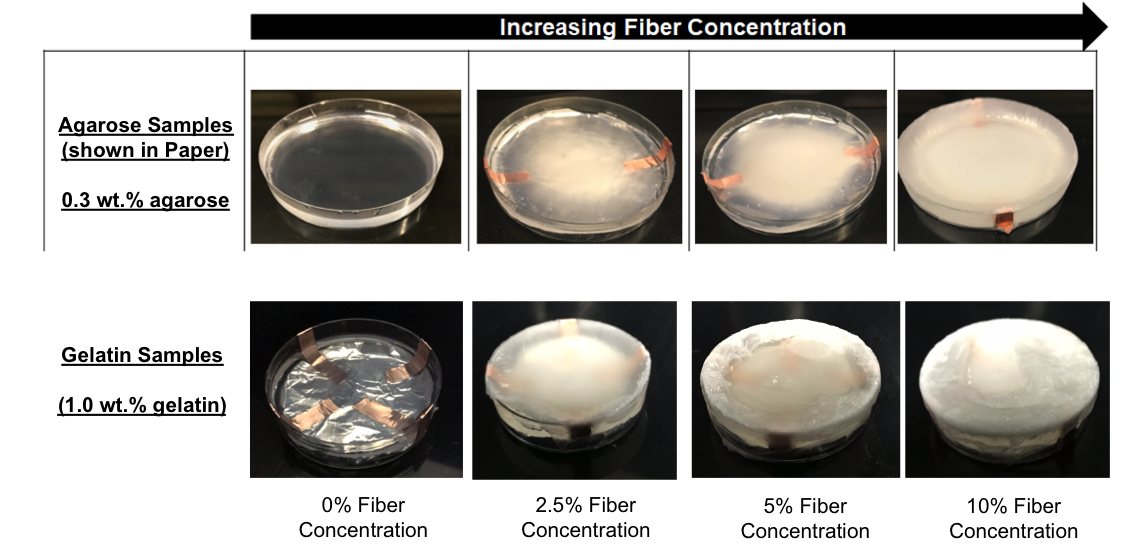


**Supplementary Figure 3.** Unmodified use of the manufacturing set-up to produce fiber-reinforced hydrogels using 1% gelatin (bottom row) instead of agarose (top row directly from Figure 5 in the paper).
